# First Principles Study of Penta-siligraphene as High-Performance Anode Material for Li-Ion Batteries

**DOI:** 10.1186/s11671-019-3097-5

**Published:** 2019-07-30

**Authors:** Hewen Wang, Musheng Wu, Zhengfang Tian, Bo Xu, Chuying Ouyang

**Affiliations:** 1 College of Chemistry and Chemical Engineering, Hubei Key Laboratory for Processing and Application of Catalytic Materials, Huanggang Normal University, Huanggang, 438000 People’s Republic of China; 2 Department of Physics, Laboratory of Computational Materials Physics, Jiangxi Normal University, Nanchang, 330022 People’s Republic of China

**Keywords:** Silicon-carbon materials, Penta-siligraphene, Anode, Li-ion batteries, First principles study

## Abstract

From first principles calculations, a novel pentagonal Si/C complexity is predicted to have potential applications as a promising anode material for Li-ion batteries. It is found that the structural and thermal stability of the penta-siligraphene (P-Si_2_C_4_) is better than penta-graphene that is composed of C atoms only. Electronic band structure analysis shows that the empty C-2*p*_*z*_ state in the P-Si_2_C_4_ provides space to accommodate and stabilize electrons from Li, which makes Li storage energetically favorable. As a result, four Li atoms can be stored by one formula unit of the P-Si_2_C_4_, corresponding to a theoretical gravimetric Li storage capacity of 1028.7 mAhg^−1^. The metallic electronic structures of the Li-adsorbed P-Li_*x*_Si_2_C_4_ as well as very small Li migration energy barriers are beneficial for fast charge/discharge performance of the battery. The mechanism on the Li adsorption interaction on the P-Si_2_C_4_ is discussed. These results demonstrate a novel strategy to design two-dimensional Si/C complex anode materials for high-performance Li-ion batteries.

**Electronic supplementary material:**

The online version of this article (10.1186/s11671-019-3097-5) contains supplementary material, which is available to authorized users.

## Background

The relatively low energy density of the currently commercialized Li-ion batteries (LIBs) is difficult to meet the requirement of the commercial electric vehicles (EVs) and becomes a big challenge for the development of the EV industry [1, 2]. To enhance the energy density of the LIBs, we need to improve the capacity of the electrode materials. Due to its very good cycling performance, graphite is the most widely used anode material, but its theoretical gravimetric capacity (372 mAhg^−1^) is relatively low [3, 4]. On the other hand, silicon has extremely high theoretical gravimetric capacity of about 4200 mAhg^−1^ [5], but the cycling performance is poor due to its very large volume expansion up to 420% at fully lithiated state [6]. To take advantages of both silicon and carbon anodes, designing a Si/C complex anode is both academically as well as technologically significant.

Siligraphene, which is a two-dimensional (2D) graphene-like layered material with C atoms partially replaced by Si atoms, was first predicted to be a stable 2D material from first principles calculations [7–10] and had been prepared successfully from experiments [11, 12]. Lin et al. had shown that 2D SiC sheets can be prepared through solution exfoliation techniques [11]. They also successfully prepared quasi-2D SiC_2_ sheets which can be preserved in air for months [12]. Later, first principles calculations suggest that siligraphene is a promising anode material that offers a theoretical capacity of 1520 mAhg^−1^ and 1286 mAhg^−1^ for g-SiC_5_ and g-SiC_2_, respectively [13]. It is shown that the siligraphene anode inherits the high cycling stability from graphite anodes as well as high capacity from silicon anodes. The predicted high Li storage capacity is attributed to the enhanced Li adsorption interaction with the siligraphene monolayer, which is related to the changes of Si atom from *sp*^2^ to *sp*^3^-like [13]. However, the electronic configuration change from *sp*^2^ to *sp*^3^-like is accompanied by obvious structural changes during Li adsorption on the siligraphene. This is not good for the cycling performance of the siligraphene as an anode material for LIBs. The better solution is to design Si/C complex materials that already have *sp*^3^-like electronic configuration.

Carbon has many types of allotropes, which are formed with *sp*, *sp*^2^, and *sp*^3^ hybridization or their combinations. The very stable *sp*^2^ plus big π-bond electronic configuration in graphite carbon is responsible for the weak Li adsorption interactions on graphene. Upon Li adsorption on monolayer graphene, charge transfer occurs from Li to the graphene layer [14]. Then, Li becomes positively charged and is binding to the graphene layer by the attractive Coulomb interaction. However, the excess charge from Li on the graphene layer breaks the big π-bond of the graphene, which is energetically unfavorable. As a result, the Li adsorption on single-layer graphene is not favored with adsorption energy of negatively higher than the cohesive energy of body-centered face (*bcc*) phase Li metal, which is not allowed in Li-ion batteries. As a result, Li storage with pristine graphene monolayer is not allowed [15]. Alternatively, hard carbon materials offer much higher Li/Na storage gravimetric capacity compared with that of graphite carbon materials [16–18]. Hard carbon material is known as an amorphous phase that is composed of both *sp*^2^ and *sp*^3^ carbon atoms [19]. Is it possible that the higher Li storage gravimetric capacity of hard carbon materials is related to the *sp*^3^ electronic configuration?

Penta-graphene, which is known as an *sp*^2^-*sp*^3^ hybrid 2D carbon allotrope [20], was predicted to be a promising anode material for Li/Na-ion batteries from first principles calculations [21]. As a 2D carbon allotrope, penta-graphene has a much stronger Li adsorption behavior compared with the conventional graphene with honeycomb structure. Is this different Li adsorption behavior also related to the *sp*^3^-like electronic configuration in penta-graphene? If the answer is yes, what is the intrinsic mechanism behind it?

Although penta-graphene is predicted to be a dynamically stable carbon allotrope, its cohesive energy is significantly higher compared with the globally most stable phase (graphite or graphene). The cohesive energy of penta-graphene is about 0.9 eV per atom higher than that of single-layer hexagonal graphene [20, 22], which makes it very difficult (if possible) for large-scale fabrication of the penta-graphene industrially. However, as for applications as anode material, large-scale fabrication is very important. Note that buckling is found in silicene and thus Si is more stable with *sp*^3^-like hybridization than *sp*^2^ [23–25] while C atoms prefer *sp*^2^ hybridization in 2D structures; it is reasonable to speculate that replacing the *sp*^3^-like C atoms with Si atoms in the structure of penta-graphene will be energetically favored. We call this structure as penta-siligraphene. Recent experiments have demonstrated that pentagonal Si-based nano-ribbons can be grown on Ag (110) [26], showing that the formation of Si-based pentagonal structure is experimentally possible.

Theoretically, the electronic and bonding nature of the penta-siligraphene (P-SiC_2_) was studied by Lopez-Bezanilla et al. and they found that P-SiC_2_ exhibits a partial inversion of the vertical ordering of the p-p-σ and p-p-π electronic bands [27]. Later, the electronic transport properties of the P-SiC_2_ are studied and compared with penta-graphene and penta-CN_2_ [28]. Interestingly, it is demonstrated that the electronic transport performance of the P-SiC_2_ can be tuned through strain engineering, and it was predicted that uniaxial compressive strain is capable of enhancing the hole mobility of monolayer penta-SiC_2_ up to 1.14 × 10^6^ cm^2^ V^−1^ s^−1^ [29]. Despite the similarity in structures, the penta-siligraphene has different transport properties compared with penta-graphene. It was found by Hu et al. that the thermal conductivity of penta-graphene exhibits standard monotonic reduction by stretching, while penta-SiC_2_ possesses an unusual non-monotonic up-and-down behavior [30]. These interesting properties of the penta-siligraphene are strongly related to the electronic and chemical nature of the Si atoms in the structure. It was also found that Si element itself is beneficial to enhance the Li adsorption, as the Li adsorption interaction on silicene is much stronger compared with that on graphene [31, 32]. Therefore, it might be interesting to know if penta-siligraphene can be used as anode materials for LIBs.

In this work, we investigate Li-ion storage behaviors in penta-siligraphene with first principles calculations, and the mechanism on how Li ion can be stored by the penta-siligraphene is specifically discussed. We start our study from thermodynamic stability of the penta-siligraphene, followed by a detailed analysis of the intrinsic interactions of Li adsorption on it. Finally, the performance of the penta-siligraphene as an anode material for LIBs is discussed.

## Computational Methods

All calculations in this work are performed using the Vienna Ab initio Simulation Package (VASP) [33] based on density functional theory (DFT). The projector augmented wave (PAW) method [34, 35] combined with the general gradient approximation (GGA) exchange and correlation functionals parameterized by Perdew-Burke-Ernzerhof (PBE) are used [36]. The cutoff energy for the plane waves is chosen to be 450 eV for all calculations. The lattice parameters and the ionic positions are fully relaxed, and the final forces are converged to 0.02 eV/Å. The electronic band structure is calculated with Heyd-Scuseria-Erznerhof (HSE06) hybrid functional [37], as hybrid functional has more accurate description of the electronic structure. The calculation of the density of states (DOS) is smeared by the Gaussian smearing method with a smearing width of 0.05 eV. The Monkhorst-Pack [38] *k*-point sampling is used and the density of the *k*-mesh is thicker than 0.05 Å^−1^ for ab initio molecule dynamics (AIMD) simulation and 0.03 Å^−1^ for other calculations. The atomic charge distribution is analyzed with the Bader charge analysis [39]. The Li-ion migration pathway is optimized with the climbing image nudged elastic band (CINEB) method [40]. The adsorption energy *E*_ad_ is calculated by:

$$ {E}_{\mathrm{ad}}=\left({E}_{\mathrm{host}+n\mathrm{Li}}-{E}_{\mathrm{host}}-{nE}_{\mathrm{Li}}\right)/n $$

where *E*_host_, *E*_Li_, and *E*_host + Li_ are the total energies of the host penta-siligraphene materials, the Li atom, and the Li-adsorbed hosts, respectively, *n* denotes the number of Li ions adsorbed on the penta-siligraphene. The influence of the van der Waals (vdW) interactions to the adsorption energy is tested using the DFT-D3 method with Becke-Jonson damping [41]. In addition to the adsorption energy, the average Li intercalation potential (vs Li^+^/Li) can be obtained directly from the difference of the adsorption energy and the cohesive energy of the Li metal (bcc phase) from *V*_ave_ =  − (*E*_ad_ − *E*_Li − cohesive_), if we choose eV and V as the units for energy and the potential, respectively.

## Results and Discussions

### Structure and Stability of Penta-siligraphene

**Fig. 1:**
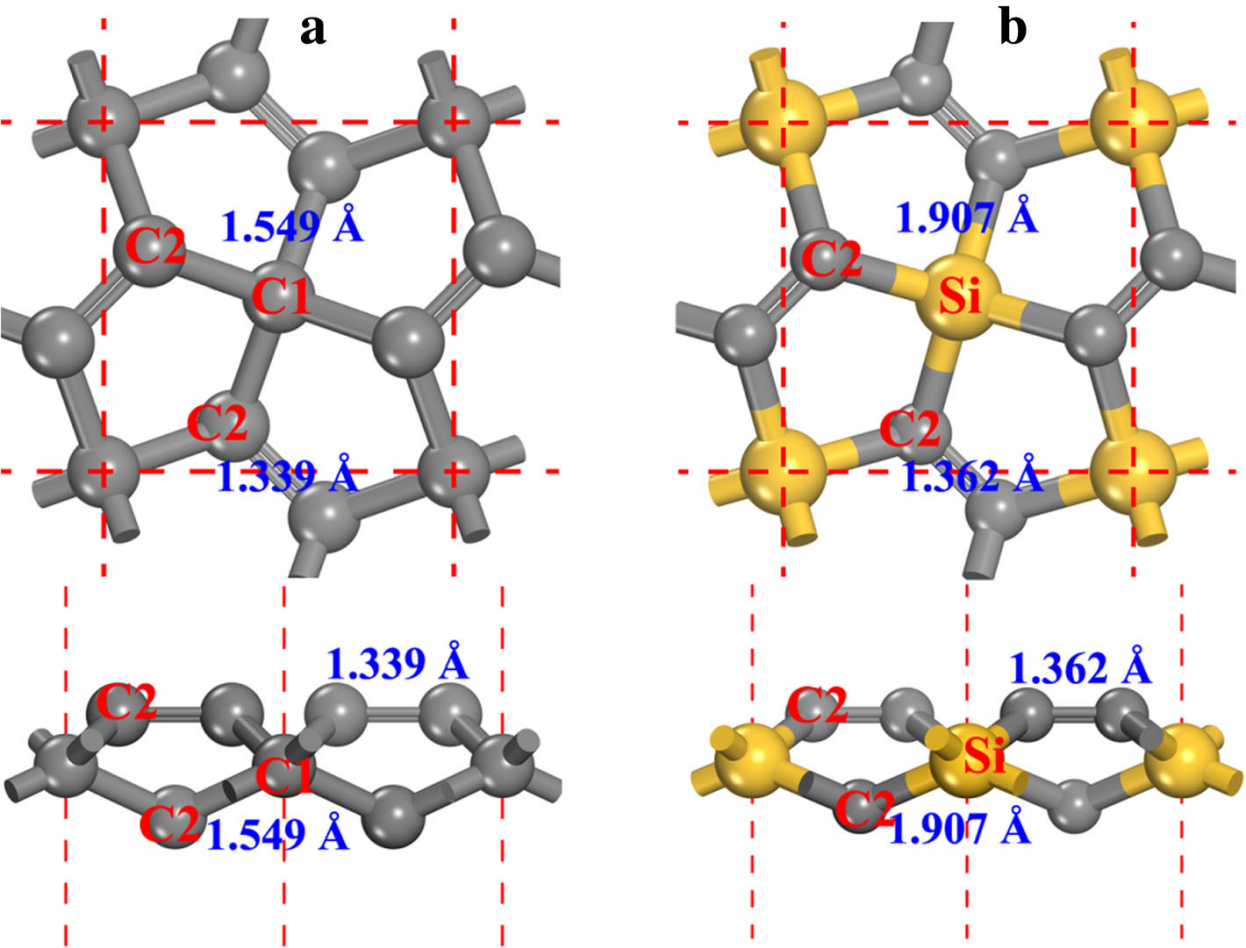
**a** The ball and stick model of penta-graphene and **b** penta-siligraphene. Both top views (up) and side views (down) are presented. The gray and yellow spheres are C and Si atoms, respectively. The 4- and 3-coordinated carbon atoms are denoted as C1 and C2, respectively. The bond lengths are also presented alongside each bond

The structure of the penta-graphene (see Fig. 1a, denoted as P-C_6_ in the following of this paper) possesses the P-42_1_ *m* symmetry (space group No. 113). The optimized lattice constants are *a* = *b* = 3.636 Å, in agreement with previous results [20, 21]. Two types of carbon atoms can be found in the structure, namely, 4-coordinated carbon (denoted as C1 in Fig. 1a) and 3-coordinated carbon (denoted as C2 in Fig. 1a). From the local geometry of the carbon atoms, we can see that C1 is *sp*^3^-like hybridized while C2 is *sp*^2^-like hybridized. Although C2 atom is regarded as *sp*^2^-like hybridized [20], the double C2–C2 bond feature makes the chemical character of the C2 atom different from that of graphene, which will be discussed in details in the following of this paper. Replacing C1 atoms with Si atoms in the P-C_6_ structure, the penta-siligraphene is formed (see Fig. 1b, the crystalline information file of the optimized structure is given in Additional file 1: SI-1 of the supplementary material) and denoted as P-Si_2_C_4_ in the following of this paper. As the atomic radius of Si atom is larger than that of C atom, the lattice constants of the P-Si_2_C_4_ (*a* = *b* = 4.405 Å) is larger than that of P-C_6_, while in good agreements with the other reported results [27–30].

**Fig. 2:**
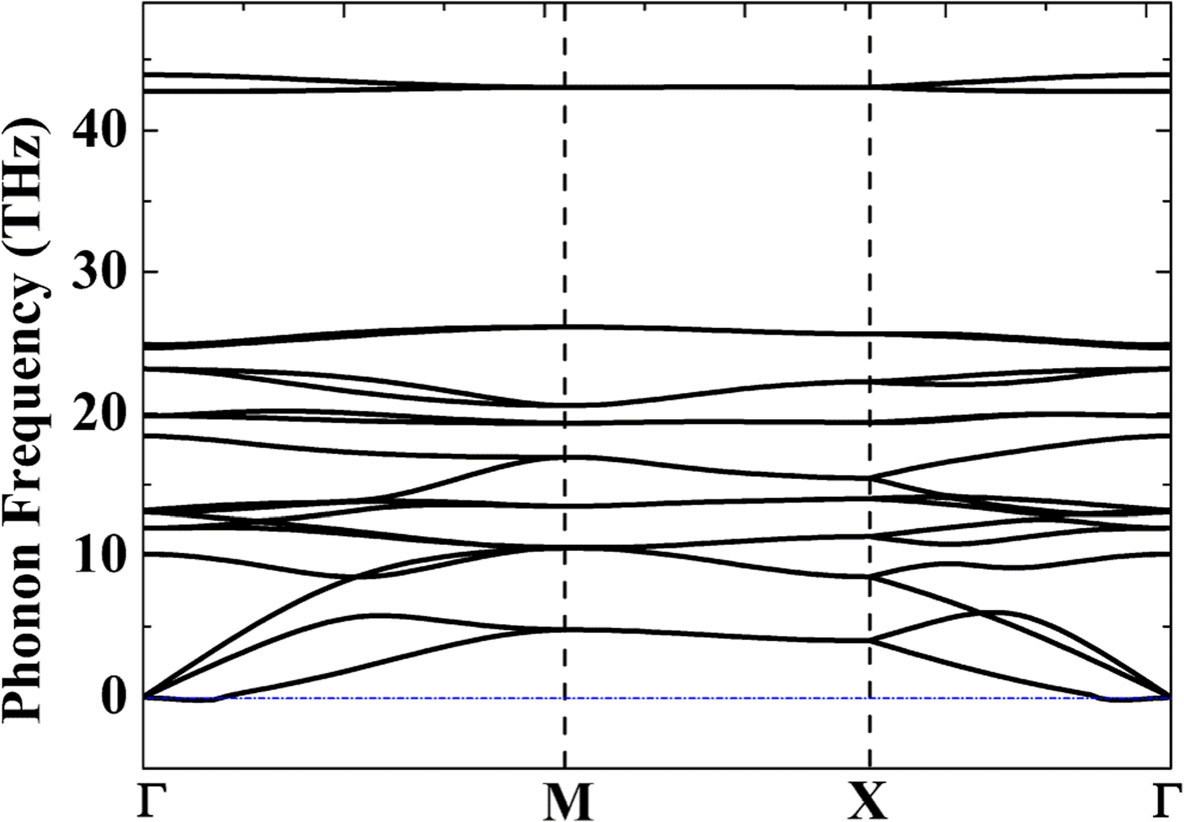
Phonon dispersion curves of the 2D P-Si_2_C_4_ monolayer calculated from linear response theory

In order to evaluate the relative thermodynamic stability, Table 1 presents the cohesive energies of different allotropes of C, Si, and C/Si complexes. Although penta-graphene (P-C_6_) is shown to be stable at 1000 K from ab initio molecular dynamics (AIMD) simulations [20], the cohesive energy of P-C_6_ (− 8.24 eV·atom^−1^) is much higher compared with that of single-layer graphene (− 9.14 eV·atom^−1^). This shows that mass production of P-C_6_ must be very difficult. On the other hand, the cohesive energy of P-Si_2_C_4_ (− 7.26 eV·atom^−1^) is only 0.2 eV higher compared with its most stable allotrope g-Si_2_C_4_ (− 7.46 eV·atom^−1^), showing that preparation of penta-siligraphene can be much easier compared with P-C_6_. To verify the structural stability of the P-Si_2_C_4_, phonon dispersion curves of the P-Si_2_C_4_ were calculated and presented in Fig. 2. Although small imaginary frequencies are found in a small region near the Γ-point (0.0039 THz or 0.13 cm^−1^), we can still believe that the system is dynamically stable, because it is generally accepted that these small imaginary frequencies (no greater than 1 cm^−1^) could be an artifact of the simulation [42]. Imaginary frequencies have also been reported in other dynamically stable 2D materials such as germanene [43] and arsenene [44]. Applying technique treatments like increasing the accuracy of the calculation or using different calculation method, these imaginary frequencies can be removed.

**Table 1:** The lattice parameters and cohesive energies of different C/Si materials

	*a* (Å)	*d*_C2–C1/Si_(Å)	*d*_C2–C2_ *d*_Si–Si_ (Å)	*E*_coh_ (eV·atom^−1^)
Graphene	2.460	1.420		− 9.14
P-C_6_	3.636	1.549	1.339	− 8.24
Diamond	3.574	1.547		− 9.01
Silicon	5.471	2.369		− 5.34
g-Si_2_C_4_	5.007	1.794	1.441	− 7.46
P-Si_2_C_4_	4.405	1.907	1.362	− 7.26

Furthermore, AIMD simulation is also performed to evaluate the structural stability of P-Si_2_C_4_ at high temperatures. AIMD are performed using 3 × 3 and 4 × 4 supercells in a canonical ensemble at temperatures of 1000 K, 1500 K, 2000 K, and 2500 K (see Additional file 1: Figure S1). Additional file 1: Figure S2 and S3 present the atomic configurations of the P-Si_2_C_4_ at the end of AIMD simulations at different temperatures using 3 × 3 and 4 × 4 supercells, respectively. As is shown, the pentagonal atomic rings are kept unchanged when the temperature is as high as 2000 K during the 20-ps simulation time, showing that the structure can bear a temperature as high as 2000 K. On the other hand, serious structural deformations are observed and hexagonal rings (see Additional file 1: Figure S2d) as well as other defects (Additional file 1: Figure S3d) appeared in the snapshots, indicating that the structures are destroyed at 2500 K. The hexagonal rings found in P-Si_2_C_4_ at 2500 K indicate that g-Si_2_C_4_ (which is composed of hexagonal rings [13]) is more stable than the penta-phase P-Si_2_C_4_, consistent with the cohesive energy given in Table 1. These results confirm that the structural stability of the P-Si_2_C_4_ is much more stable compared with that of the P-C_6_, which can only bear a temperature of 1000 K.

### Li Adsorption on Penta-siligraphene

**Fig. 3:**
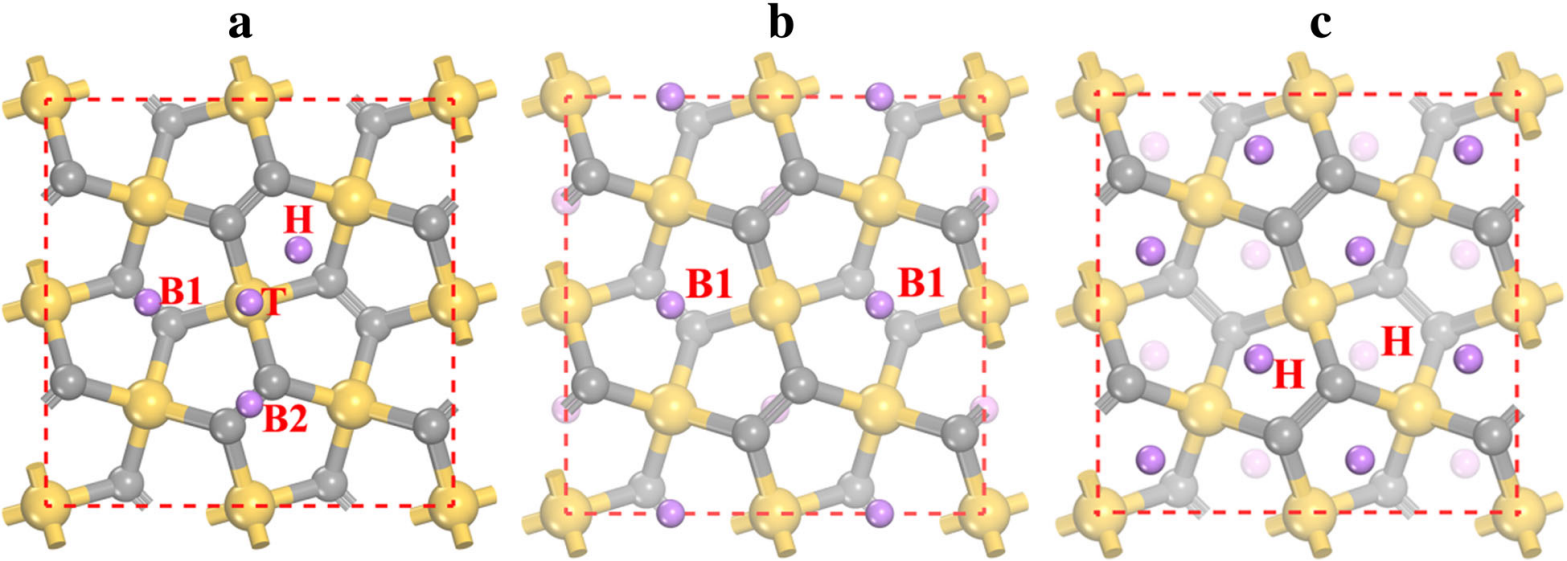
Li adsorption sites on **a** penta-siligraphene surface and the atomic configuration of the most stable structures of **b** Li_2_Si_2_C_4_ and **c** Li_4_Si_2_C_4_. The yellow (largest), gray (middle sized), and purple (smallest) spheres are Si, C, and Li atoms, respectively. H, T, B1, and B2 denote the Li adsorption sites

In order to study the Li adsorption on the penta-siligraphene P-Si_2_C_4_, different Li adsorption sites are considered and four stable adsorption sites (as shown in Fig. 3a) can be found after relaxation. The stable Li adsorption sites are top site of Si atom (denoted as T), hollow site (denote as H) of the Si_2_C_3_ pentagon ring, and bridge sites between two C2 atoms at the down-layer (B1) and up-layer (B2). The preference of the Li-ion adsorption on these sites can be characterized by the adsorption energies presented in Table 2. The results show that the most stable Li adsorption site is the B1 site, with adsorption energy of − 1.922 eV. On the other hand, the adsorption energy at the H site (− 1.905 eV) is very close to the B1 site. The Li adsorption energies are also represented by the adsorption heights, as lower adsorption energy corresponds to smaller adsorption heights (see Table 2). At the beginning of the Li adsorption process, Li ions are preferred to stay at the most stable B1 sites. After all B1 sites are occupied (corresponding to a stoichiometry of Li_2_Si_2_C_4_ and see Fig. 3b), Li ions start to stay at H sites. As the distance between B1 and H site is very small (~ 1.5 Å), strong repulsion interaction occurs to the Li ions at B1 and H sites. As a result, Li ions at B1 sites are repulsed to the nearby H sites and therefore B1 sites become empty while all H sites are occupied at the state of Li_4_Si_2_C_4_ (see Fig. 3c). The influence of the vdW interaction to the Li adsorption energy is also tested, and the results are given in the parentheses in Table 2. As is shown, vdW interaction contributes from − 0.12 to − 0.17 eV to the adsorption energy for different adsorption sites, showing that vdW interaction is in favor of Li adsorption.

**Table 2:** The Li adsorption energies/heights at different sites on the unit cell of P-Si_2_C_4_. The adsorption height is defined as the vertical distance from the Li ion to the P-Si_2_C_4_ center plane. The numbers in the parentheses are corresponding values with vdW corrections

	H site	T site	B1 site	B2 site
*E*_ad_ (eV)	− 1.905(− 2.048)	− 1.732(− 1.903)	− 1.922(− 2.053)	− 1.713(− 1.834)
Height (Å)	2.066(2.064)	2.241(2.233)	1.629(1.627)	2.670(2.654)

**Fig. 4:**
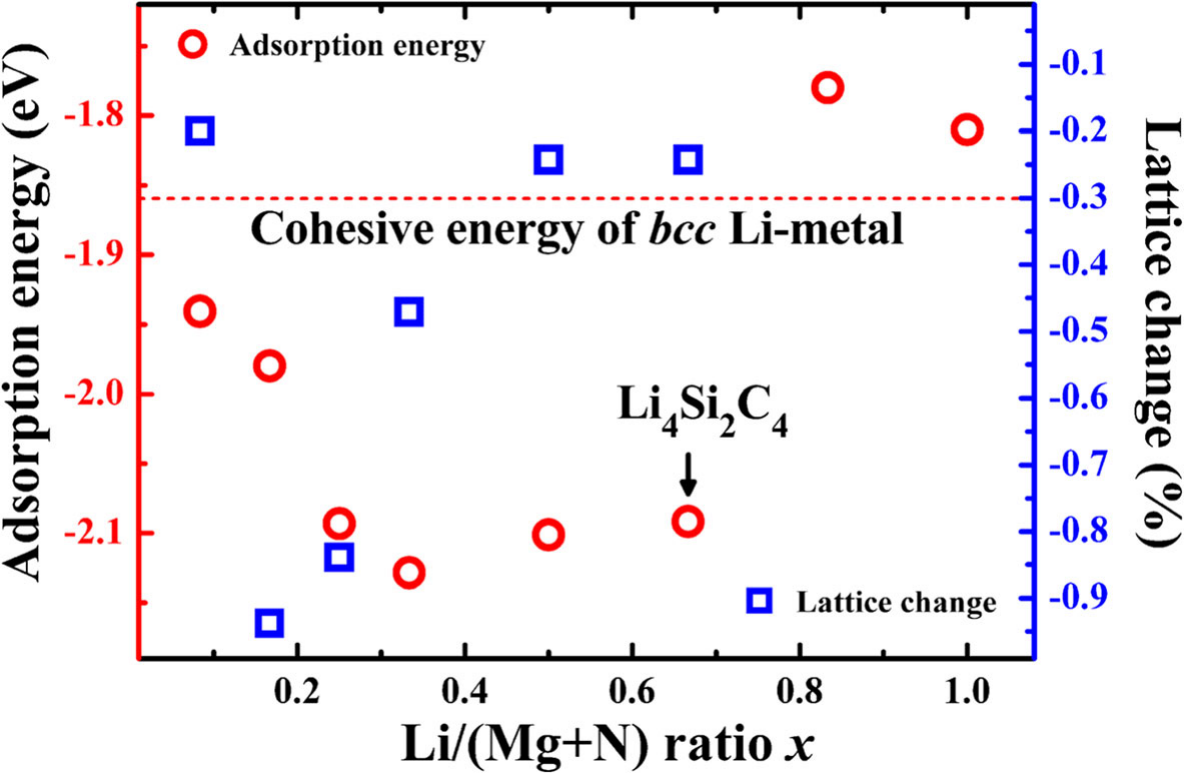
The calculated Li adsorption energies on P-Si_2_C_4_ surface (red cycles, left-hand axis) and the lattice constant change (blue squares, right-hand axis) as a function of the Li adsorption concentration (Li/(C+Si) ratio). The cohesive energy of *bcc* phase Li metal is also included for comparison

Comparing the Li adsorption energies with the cohesive energy of the *bcc* phase Li metal (− 1.86 eV·atom^−1^), we can judge that Li adsorption is electrochemically active or not. If the adsorption energy is lower than the cohesive energy of Li metal, Li-ion adsorption is favored and the adsorption corresponds to a positive discharge potential. As given in Table 1, the adsorption energies on both B1 and H sites are lower than − 1.86 eV, showing that both B1 and H sites are electrochemically active sites for Li storage. To evaluate the Li storage capacity, the Li adsorption energies at different Li-ion concentrations *x* (Li/(Si + C) ratio) are calculated and compared with the cohesive energy of *bcc* Li metal. As it is shown in Fig. 4, the Li adsorption energies are lower than − 1.86 eV when the Li/C ratio *x* is smaller than 2/3, corresponding to 4 Li atoms adsorbed in one unit cell of the P-Si_2_C_4_ and a theoretical gravimetric capacity of 1028.7 mAhg^−1^ (Li_4_Si_2_C_4_). The energy density of a battery, which is equal to the capacity times the output voltage, is more concerned compared with the Li storage capacity. A good anode material should have relatively low electrochemical potentials, which can be obtained from the adsorption energies. The average potential is about 0.1–0.2 V, which is relatively low and beneficial to a higher output voltage of a full battery system. Furthermore, Fig. 4 also presents the lattice constant changes at different Li adsorption concentrations. As is shown, the lattice constant of the P-Si_2_C_4_ shrinks slightly upon Li adsorption. When the concentration *x* is 1/6, the lattice change reaches the largest value, which is as small as − 0.94%, indicating that the volume change will be very small during the charge/discharge process. The small volume change is beneficial to maintain the structure of the P-Si_2_C_4_ to be stable during the cycling.

### Electronic Structure Analysis of the Penta-siligraphene upon Li Adsorption

**Fig. 5:**
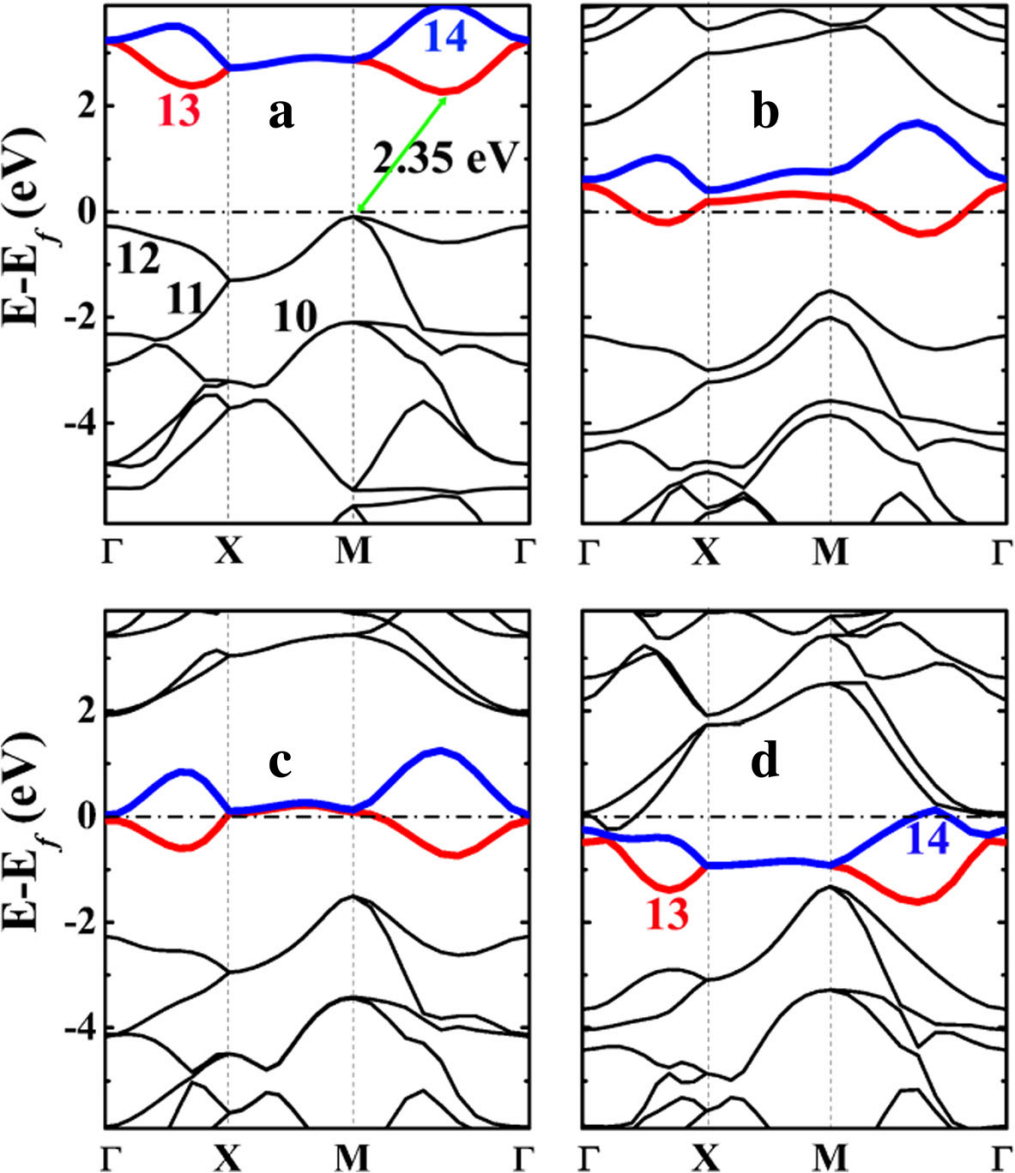
The electronic band structures of **a** penta-siligraphene P-Si_2_C_4_ and its lithiated states **b** P-LiSi_2_C_4_, **c** P-Li_2_Si_2_C_4_, and **d** P-Li_4_Si_2_C_4_ calculated from HSE06. The Fermi level is selected to be 0 eV. The numbers from 10 to 14 in **a** and **d** denote the number of the band, while the band numbers 13 and 14 are highlighted with red and blue colors, respectively. The labeling of bands is conformed to the VASP code, in which band labeling is referenced to the valence and conduction bands and the core electrons are not included in the labeling

The electronic band structures of the penta-siligraphene (P-Si_2_C_4_) and its lithiated states are presented in Fig. 5. As is seen, P-Si_2_C_4_ is a semiconductor with an indirect band gap of about 2.35 eV, which is much smaller compared with 3.46 eV of the penta-graphene P-C_6_ (see Additional file 1: Figure S4). The smaller band gap of P-Si_2_C_4_ than P-C_6_ is originated from the enhanced dispersion of the highest occupied bands (Nos. 11 and 12 in Fig. 5a), particularly at the high symmetry M and Γ points. The band gap is opened between the energy bands of No. 12 (the highest occupied band) and No. 13 (the lowest unoccupied band). The energy level of band No. 12 is raised up substantially at the M point, which raises the Fermi level and in turn decreases the band gap.

**Fig. 6:**
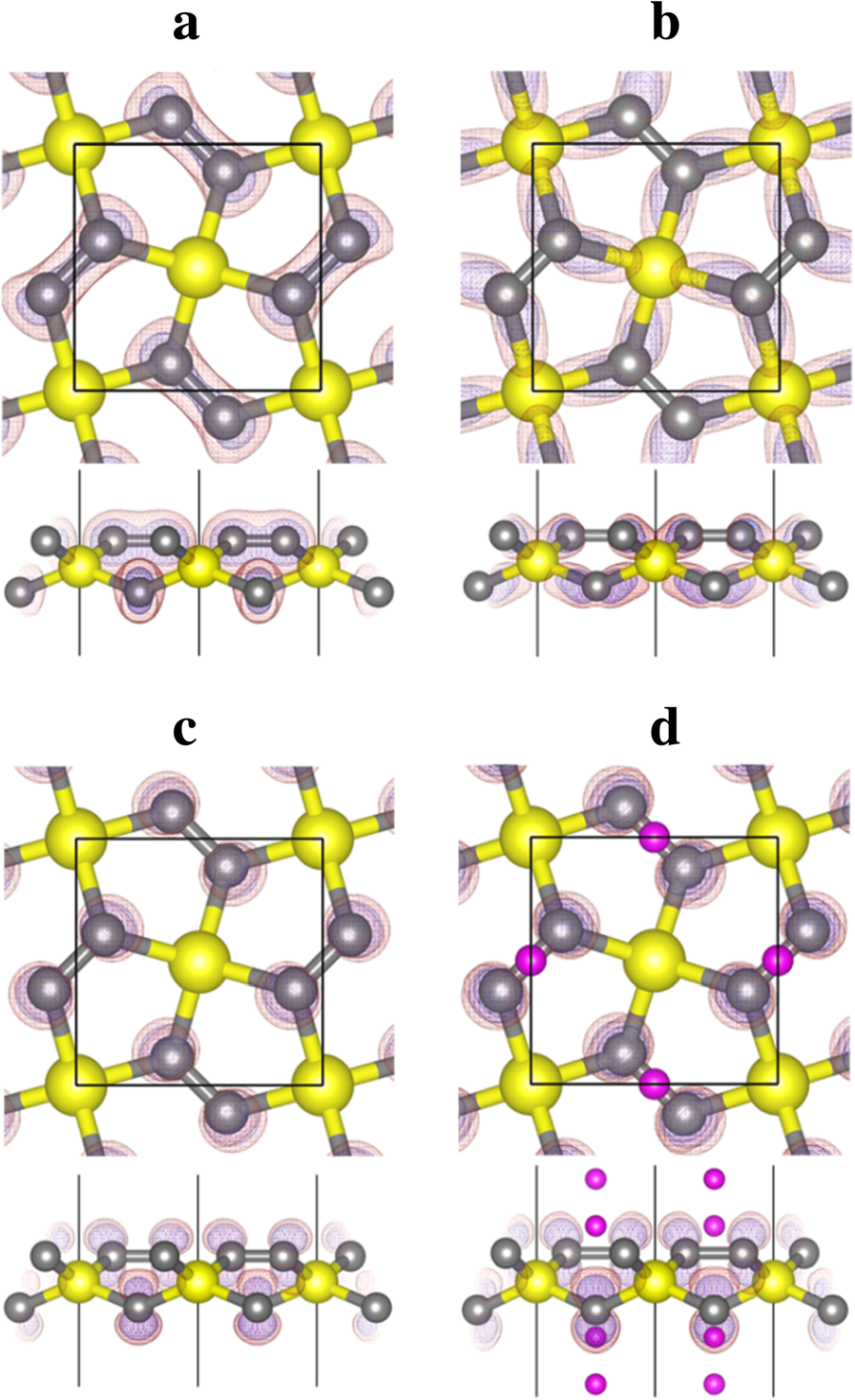
Band decomposed charge density contours for bands **a **No. 10, **b** No. 12, and **c** No. 13 of the penta-siligraphene (P-Si_2_C_4_, Fig. 5a) and band **d** No. 13 of the lithiated penta-siligraphene (Li_4_Si_2_C_4_, Fig. 5d). The yellow (large), gray (middle-sized), and purple (small) spheres are Si, C, and Li atoms, respectively. The charge density contours are displayed with transparent red (with isosurface value of 0.02 e/Å^3^) and blue (with isosurface value of 0.01 e/Å^3^) colors

From the analysis of the shape of the charge density (wavefunction) projected to bands No. 10–14 shown in Fig. 6, we can see that band No. 12 corresponds to the bonding states of the σ-bonds formed between C and Si atoms, while band No. 13 (and 14) corresponds to the 2-*p*_z_ state of the C atom. The empty C-*p*_z_ state provides space to accommodate and stabilize electrons from Li adsorption, which makes Li adsorption process energetically favorable.

The enhanced dispersion of the highest occupied energy bands in P-Si_2_C_4_ can be attributed to two factors: firstly, the Coulomb attraction between the electrons occupying band No. 12 (C–Si σ-bonds) and the positively charged Si atom, compared with that in carbon-only penta-graphene P-C_6_. Bader charge analysis shows that Si atom is positively charged in the penta-siligraphene P-Si_2_C_4_. As shown in Table 3, the Bader charge of Si atoms in either siligraphene or penta-siligraphene is about 1.65 **e**, showing that Si atoms are positively charged with + 2.35 **e**. On the other hand, C1 atom in penta-graphene (P-C_6_) is also positively charged, but the net charge is only + 0.08 **e**. Therefore, in addition to the covalent bonding interaction, strong Coulomb interactions between C and Si atoms occur in P-Si_2_C_4_, compared with that in the P-C_6_. This is beneficial to the dispersion of the occupied energy bands (No. 12) near the Fermi level. Secondly, the enhanced buckling in the P-Si_2_C_4_ may also contribute to the dispersion of the band No. 12 (the bonding state of the C–Si σ-bond), as larger buckling denotes more *sp*^3^-hybridized and stronger σ-bonding are formed between the C–Si atoms. This is also an important reason for the stable structural stability of the P-Si_2_C_4_ compared with that of P-C_6_. More importantly, the buckling between the Si and C atoms increases with Li adsorption and becomes 0.876 Å at the lithiated state P-Li_4_Si_2_C_4_. In this state, the C–Si–C (102.50° and 124.59°) bond angles of the SiC_4_ tetrahedron becomes closer to 108.47° of a standard tetrahedron, showing that the *sp*^3^-hybridization of the Si atom and the strength of the C–Si bonds become stronger upon Li adsorption. Consequently, the dispersion of the band No. 12 is also enhanced with the increased Li adsorption concentration, as can be seen in Fig. 4.

**Table 3:** The Bader charge (in electrons) of penta-graphene (P-C_6_), penta-siligraphene (P-Si_2_C_4_) and their Li-adsorbed states P-Li_4_C_6_ and P-Li_4_Si_2_C_4_. Siligraphene (g-Si_2_C_4_) is also included for comparison. The values in the parentheses are the positive or negative charge of the correspond atoms

Atoms	P-C_6_	g-Si_2_C_4_	P-Si_2_C_4_	P-Li_4_C_6_	P-Li_4_Si_2_C_4_
C1/Si	3.92 (+ 0.08)	1.65 (+ 2.35)	1.64 (+ 2.36)	3.99 (+ 0.01)	1.65 (+ 2.35)
C2/C3	4.04 (− 0.04)	3.68/5.68	5.18 (− 1.18)	4.67(− 0.47)	5.83 (− 1.83)
Li	–	–	–	0.34	0.40/0.29

Upon Li adsorption on the surface of penta-siligraphene (P-Si_2_C_4_), charge transfers from the Li atoms (in real battery operation, Li^+^ ions come from the inner circuit while same amount of electrons come from the external circuit) to the carbon atoms in P-Si_2_C_4_. As a result, excess electrons will move down the unoccupied bands (bands No. 13 and 14) giving rise to metallic electronic structures of the system, as shown in Fig. 6b–d. The metallic electronic structures ensure good electronic conductivity of the P-Si_2_C_4_ anode during the charge/discharge process, which is beneficial to the rate performance of the battery system using the P-Si_2_C_4_ anode.

As discussed above and shown by the band projected charge density in Fig. 6, bands No. 13 and 14 are the *p*_z_ states of the C atom in P-Si_2_C_4_. These empty bands are very important for Li adsorption. In addition to the Coulomb attraction between the positively charged Li ions and the negatively charged P-Si_2_C_4_ substrate, the electrons occupying the C-*p*_z_ states have a strong Coulomb attraction to the positively charged Si atom (which moves down the energy levels of bands No. 13 and 14, and thus lowers the total energy of the substrate). As a consequence, Li adsorption on P-Si_2_C_4_ is energetically more favorable. As a unit cell of the P-Si_2_C_4_ contains 4 C atoms, it is expected that 4 Li atoms can be adsorbed on the P-Si_2_C_4_ surface. After the C-*p*_z_ states are completely occupied, adsorption of more Li atoms on the P-Si_2_C_4_ will be energetically unfavorable. This is in agreement with the calculated adsorption energies presented in Fig. 4.

### Li-Ion Migration Dynamics on P-Si_2_C_4_

The rate performance of the P-Si_2_C_4_ anode is determined by electronic conduction and Li-ion diffusion dynamics. As discussed above, although the electronic structure of the pristine P-Si_2_C_4_ is an insulator, it becomes metallic spontaneously upon Li adsorption, even when Li concentration is low. Therefore, the electronic conductivity should be good enough for application as an anode material. Then, the Li-ion diffusion on the penta-siligraphene becomes the rate control step. As the structure of the P-Si_2_C_6_ is similar to that of the penta-graphene (P-C_6_) on which Li ion diffuses very fast [21], it is expected that Li-ion diffusion on P-Si_2_C_6_ can also be very fast.

**Fig. 7:**
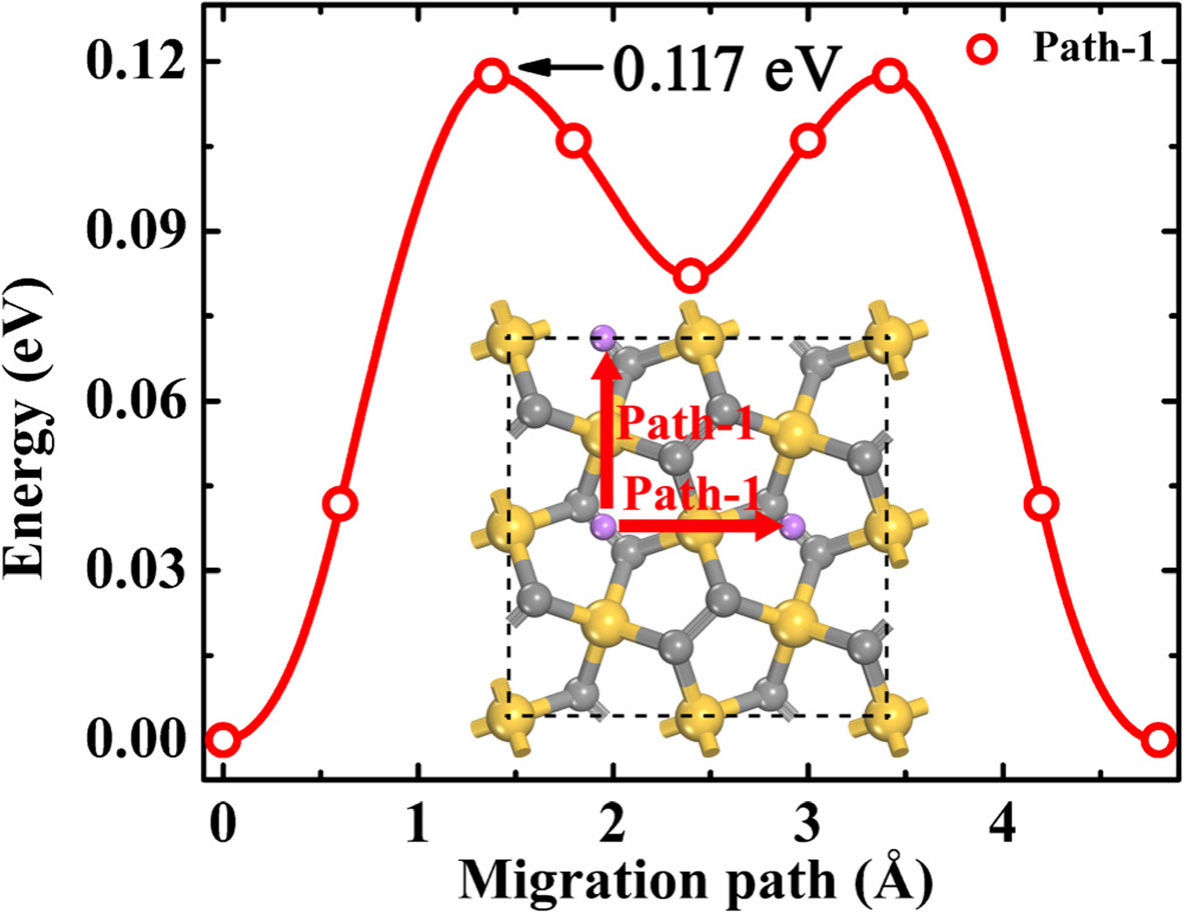
The dilute Li-ion migration pathway and the corresponding energy profile along the pathway on P-Si_2_C_4_. The gray (middle sized), golden, and purple spheres are C, Si, and Li atoms, respectively. The red arrows illustrate the two-dimensional diffusion networks

The rate performance of a battery is strongly related to the state of charge (SOC), namely, the Li-ion diffusion is dependent on the Li adsorption concentration. In order to evaluate the Li-ion diffusion dynamics at different SOC, two extreme concentrations are considered, namely dilute Li ion and dilute Li vacancy. For simulation of dilute Li-ion diffusion, one Li ion is adsorbed on a supercell of the P-Si_2_C_4_. From above discussions in the section “Li Adsorption on Penta-siligraphene,” Li ion prefers to stay at B1 site when the Li concentration is low, as shown in Fig. 7. Considering the symmetry of the structure, only one Li migration pathway (denoted as Path-1 in Fig. 7) can be found and Path-1 forms a complete 2D Li diffusion network on the P-Si_2_C_4_ surface. The dilute Li-ion migration energy barrier on P-Si_2_C_4_ along the NEB method optimized pathway is about 0.117 eV, which is smaller than that of on P-C_6_ (0.17 eV, Path-II) [21] and g-Si_2_C_4_ (0.548 eV) [13]. When the SOC becomes 50%, namely, the material is discharged into the state of Li_*2*_Si_2_C_4_, all Li ions still occupy the B1 sites (see Fig. 3b) and thus the Li-ion diffusion pathway is the same as the case of dilute Li ion. These results show that Li-ion diffusion can be very fast at the beginning half of the discharge process.

**Fig. 8:**
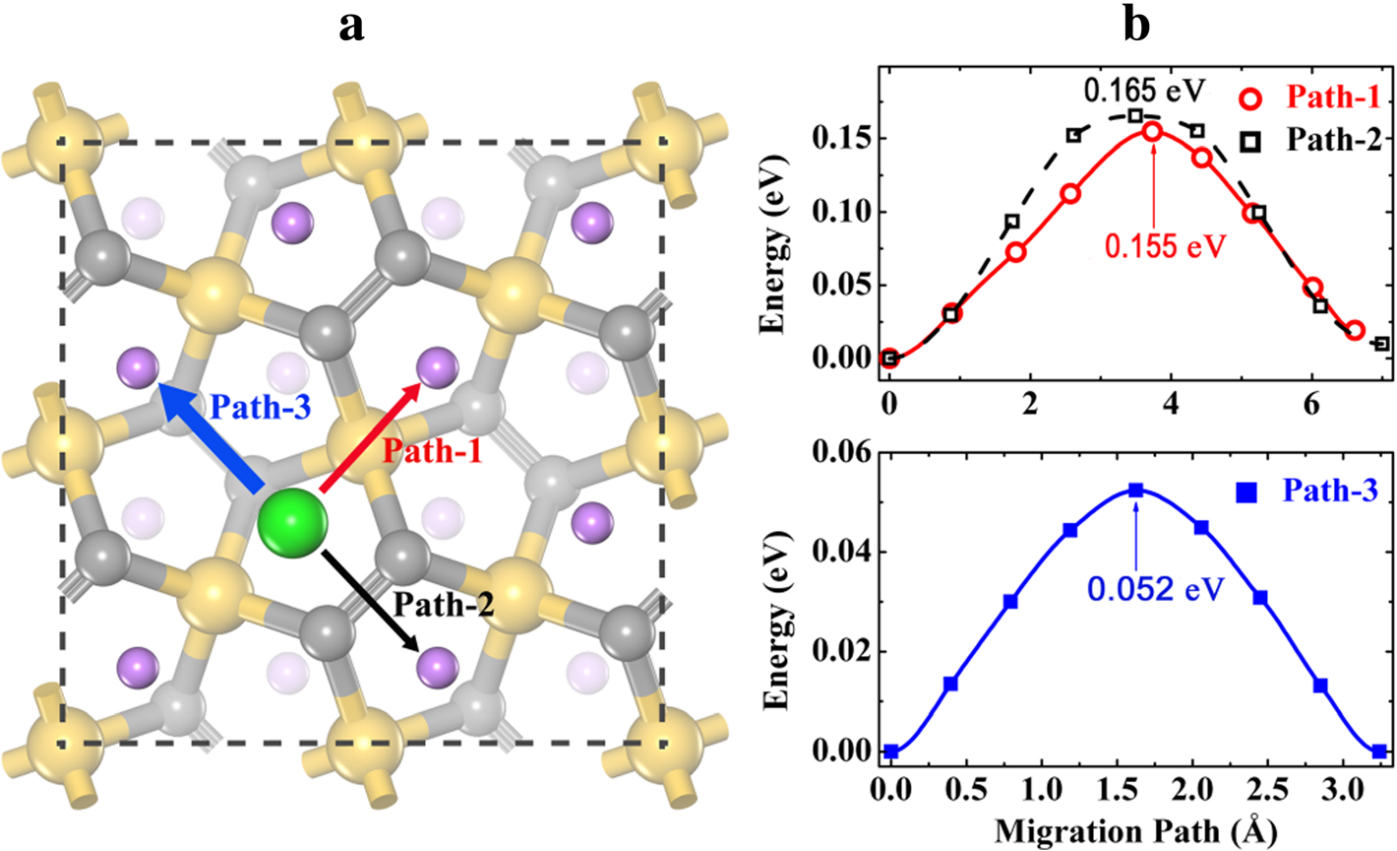
**a** Dilute Li vacancy migration pathways and **b** the corresponding energy profiles on fully lithiated P-Li_4_Si_2_C_4_. The gray (middle sized), golden, and purple spheres are C, Si, and Li atoms, respectively. The large green sphere represents the dilute Li vacancy. The thick/thin arrows indicate fast/slow migration pathways which form the two-dimensional diffusion networks

For the case of dilute Li vacancy, we remove one Li ion from the fully lithiated state Li_4_Si_2_C_4_ and create one dilute Li vacancy in the supercell. As discussed above, Li ion prefers to occupy the H site when the Li concentration is high (see Fig. 3c). Therefore, three different Li vacancy migration pathways are considered, as shown in Fig. 8a. Path-1 refers to the Li vacancy migration from the H site to its neighboring H site across the top of a Si atom (across T site). Path-2 corresponds to the pathway across the top of the middle point of the C2–C2 dimer at the top layer (across B2 site). Path-3 is the pathway along the C2–C2 dimer at the down-layer (across B1 site). The energy profiles along the optimized pathways are given in Fig. 8b. As is seen, the Li-ion migration energy barriers along these pathways are very low, particularly for Path-3 (0.052 eV). The energy profiles along Path-1 and Path-2 are slightly asymmetric, because of the large relaxation of the Li ions when one Li vacancy is created. The extremely low energy barrier along Path-3 is reasonable, since Path-3 crosses B1 site (energetically most favorable adsorption site). However, Path-3 alone is not able to form a complete Li diffusion network on the surface of the P-Si_2_C_4_. Therefore, Path-1 or Path-2 must take part in the diffusion process, and the overall energy barrier for dilute Li vacancy migration is 0.155 eV or 0.165 eV. Although higher than dilute Li-ion migration (0.117 eV), the energy barrier for dilute Li vacancy migration is also very small compared with that for P-C_6_ (0.25 eV, Path-II’) [21] and g-Si_2_C_4_ (0.233 eV) [13]. As the Li migration energy barriers in P-Si_2_C_4_ are always lower than those in P-C_6_ and g-Si_2_C_4_ (both dilute Li ion and dilute Li vacancy), it is expected that the rate performance of the P-Si_2_C_4_ is the best one among the three similar anode candidates.

## Conclusion

In summary, based on first principles calculations, we predicted that 2D pentagonal Si/C compound P-Si_2_C_4_ can be potentially used as anode materials for LIBs. Phonon dispersion data confirmed the dynamic stability of the P-Si_2_C_4_ structure at ground state, while AIMD simulation shows that the structure of the P-Si_2_C_4_ can be stable at temperatures as high as 2000 K. The unique 2D buckled pentagonal structure promotes special empty C-2*p*_*z*_ states that facilitate Li adsorption on the surface of the P-Si_2_C_4_, which offers a gravimetric Li storage capacity of 1028.7 mAhg^−1^. The calculated dilute Li-ion/Li vacancy migration energy barriers show that Li-ion diffusion on the surface of the P-Si_2_C_4_ can be faster than both the pentagonal graphene (P-C_6_) and the honeycomb-structured siligraphene. The metallic electronic structure of the lithiated P-Li_*x*_Si_2_C_4_ ensures good electronic conductivity of the material as electrodes. These advantages are crucial features to the P-Si_2_C_4_ as a promising anode material for LIBs. In summary, our first principles study offers a novel strategy to design high-performance Si/C complexity for the application in LIBs.

## Additional file

Additional file 1: Supplementary online material for “First principles study of penta-siligraphene as high-performance anode material for Li-ion batteries” (DOCX 2536 kb)

## Data Availability

All data generated or analyzed during this study are included in this published article.

## Abbreviations

2D: Two-dimensional
AIMD: Ab initio molecule dynamics
*bcc*: Body-centered face
CINEB: Climbing image nudged elastic band
DFT: Density functional theory
DOS: Density of states
EVs: Electric vehicles
GGA: General gradient approximation
HSE06: Heyd-Scuseria-Erznerhof
LIBs: Li-ion batteries
PAW: Projector augmented wave
PBE: Perdew-Burke-Ernzerhof
SOC: State of charge
VASP: Vienna Ab initio Simulation Package
vdW: van der Waals
