# Supplementary material for: First Principles Study of Penta-siligraphene as High-Performance Anode Material for Li-Ion Batteries
Source: Nanoscale Res Lett. 2019 Jul 30;14:260. doi: 10.1186/s11671-019-3097-5 (PMC6667540; doi:10.1186/s11671-019-3097-5)
Supplement: Supplementary file 1 — Supplementary online material for “First principles study of penta-siligraphene as high-performance anode material for Li-ion batteries” (DOCX 2536 kb) [file 11671_2019_3097_MOESM1_ESM.docx]

**Supplementary Material for**

**First-principles study of penta-siligraphene as high-performance anode material for Li-ion batteries**

Hewen Wang^1,2^, Musheng Wu^1*^, Zhengfang Tian^1^, Bo Xu^2^, Chuying Ouyang^1,2*^

**SI-1:** The crystallographic information file of the penta-siligraphene P-Si_2_C_4_.

#============================================================

# CRYSTAL DATA

#----------------------------------------------------------------------

data_VESTA_phase_1

_chemical_name_common 'penta-sili-relaxed'

_cell_length_a 4.40500

_cell_length_b 4.40500

_cell_length_c 15.00000

_cell_angle_alpha 90

_cell_angle_beta 90

_cell_angle_gamma 90

_space_group_name_H-M_alt 'P 1'

_space_group_IT_number 1

loop_

_space_group_symop_operation_xyz

'x, y, z'

loop_

_atom_site_label

_atom_site_occupancy

_atom_site_fract_x

_atom_site_fract_y

_atom_site_fract_z

_atom_site_adp_type

_atom_site_U_iso_or_equiv

_atom_site_type_symbol

Si1 1.0 0.499930 0.499930 0.500000 Uiso 0.012670 Si

Si2 1.0 0.000000 0.000000 0.500000 Uiso 0.012670 Si

C1 1.0 0.390610 0.109320 0.455610 Uiso 0.012670 C

C2 1.0 0.609250 0.890550 0.455610 Uiso 0.012670 C

C3 1.0 0.109320 0.609250 0.544390 Uiso 0.012670 C

C4 1.0 0.890550 0.390610 0.544390 Uiso 0.012670 C

**SI-2:** AIMD temperature variation data





Fig. S1. Temperature variation of the AIMD simulations using a NVT ensemble in a 4×4 supercell of the penta-siligraphene P-Si_2_C_4_ at 1000 K, 1500 K, 2000 K, and 2500 K.

**SI-3:** Snapshots of atomic configurations from AIMD using 3×3 supercell


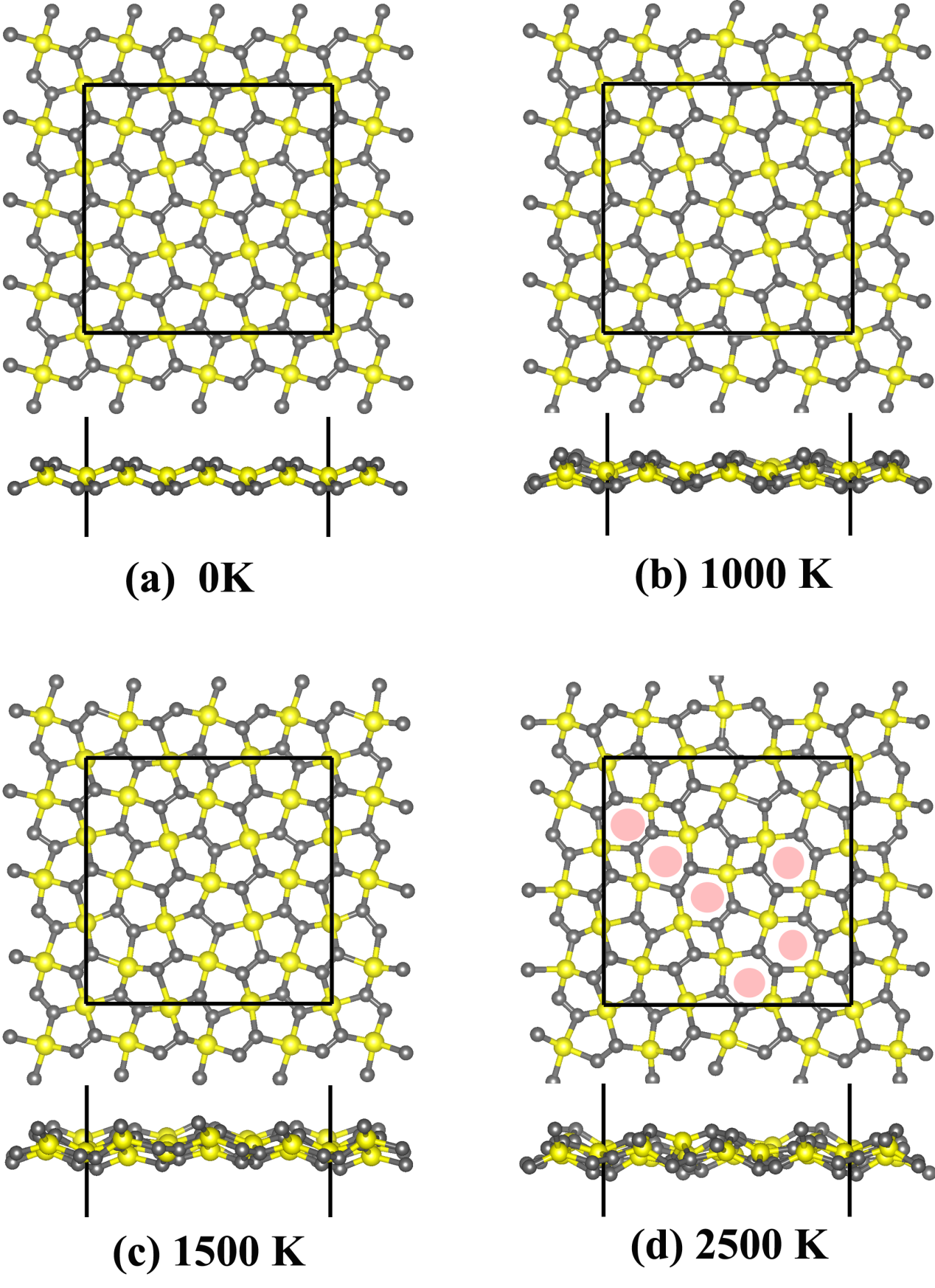


Fig. S2. Snapshots of atomic configurations of penta-siligraphene P-Si_2_C_4_ at the end of AIMD simulations using a 3×3 supercell at 1000 K (b), 1500 K (c) and 2500 K (d). For comparison purpose, (a) presents the ground state atomic structure at 0 K. The pink spherical area denotes hexagonal atomic rings formed in the supercell after AIMD simulation at 2500 K.

**SI-4:** Snapshots of atomic configurations from AIMD using 4×4 supercell


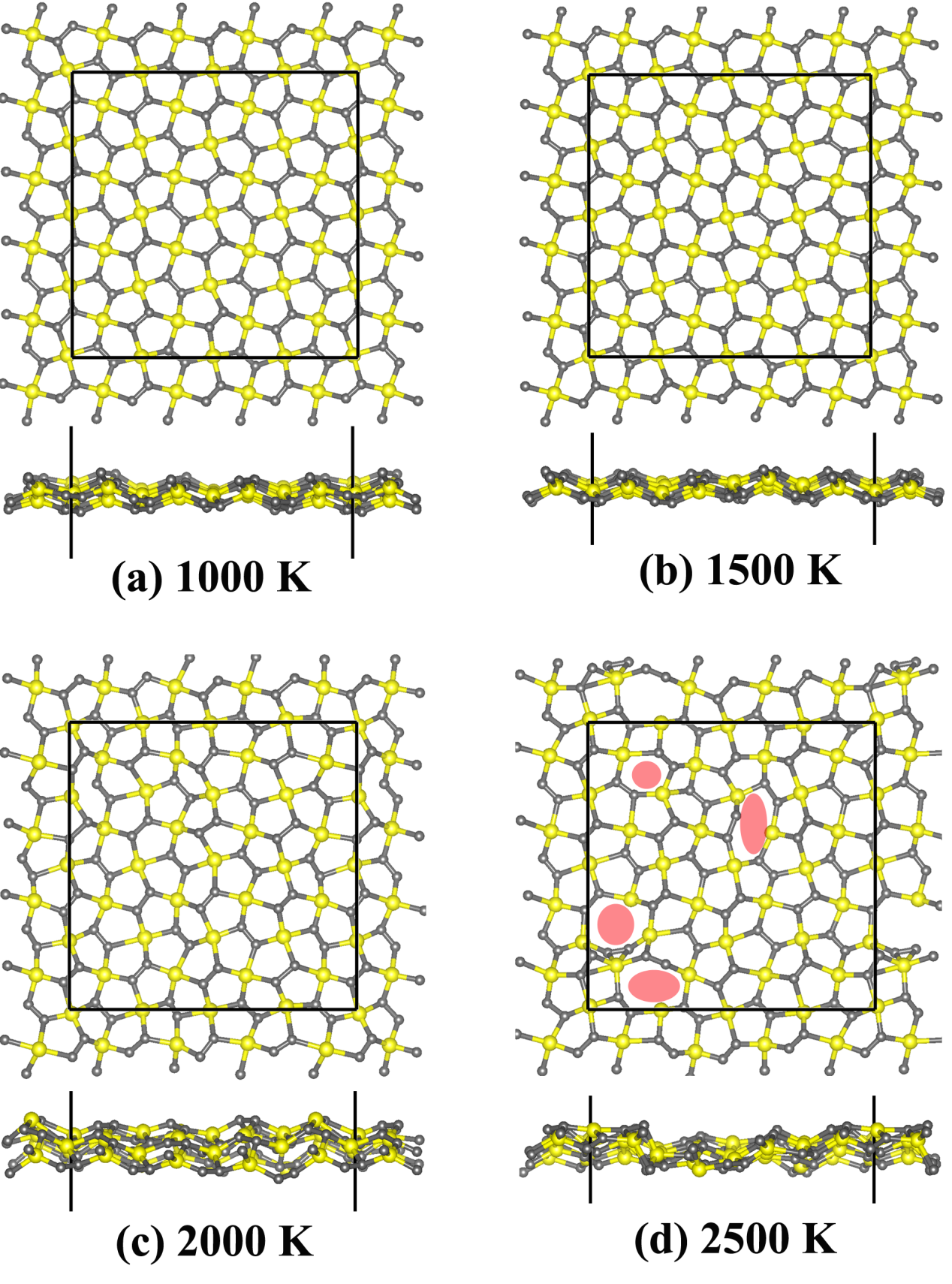


Fig. S3. Snapshots of atomic configurations of penta-siligraphene P-Si_2_C_4_ at the end of AIMD simulations using a 4×4 supercell at 1000 K (a), 1500 K (b), 2000 K (c) and 2500 K (d). The pink area denotes hexagonal atomic rings and other defects formed in the supercell after AIMD simulation at 2500 K.

**SI-5:** Electronic structures of penta-graphene and penta-siligraphene





Fig. S4. The electronic band structures of penta-graphene (a) and penta-siligraphene (b) calculated with HSE06. The Fermi level is selected to be 0 eV.
